# Phylodynamics of Enterovirus A71-Associated Hand, Foot, and Mouth Disease in Viet Nam

**DOI:** 10.1128/JVI.00706-15

**Published:** 2015-06-17

**Authors:** Jemma L. Geoghegan, Le Van Tan, Denise Kühnert, Rebecca A. Halpin, Xudong Lin, Ari Simenauer, Asmik Akopov, Suman R. Das, Timothy B. Stockwell, Susmita Shrivastava, Nghiem My Ngoc, Le Thi Tam Uyen, Nguyen Thi Kim Tuyen, Tran Tan Thanh, Vu Thi Ty Hang, Phan Tu Qui, Nguyen Thanh Hung, Truong Huu Khanh, Le Quoc Thinh, Le Nguyen Thanh Nhan, Hoang Minh Tu Van, Do Chau Viet, Ha Manh Tuan, Ho Lu Viet, Tran Tinh Hien, Nguyen Van Vinh Chau, Guy Thwaites, Bryan T. Grenfell, Tanja Stadler, David E. Wentworth, Edward C. Holmes, H. Rogier Van Doorn

**Affiliations:** a Marie Bashir Institute for Infectious Diseases and Biosecurity, Charles Perkins Centre, School of Biological Sciences and Sydney Medical School, The University of Sydney, Sydney, Australia; b Oxford University Clinical Research Unit, Ho Chi Minh City, Viet Nam; c Department of Environmental Systems Science, ETH Zurich, Zurich, Switzerland; d Swiss Institute of Bioinformatics, Lausanne, Switzerland; e J. Craig Venter Institute, Rockville, Maryland, USA; f Hospital for Tropical Diseases, Ho Chi Minh City, Vietnam; g Children's Hospital 1, Ho Chi Minh City, Vietnam; h Children's Hospital 2, Ho Chi Minh City, Vietnam; i Centre for Tropical Medicine, Nuffield Department of Medicine, University of Oxford, Oxford, United Kingdom; j Department of Ecology and Evolutionary Biology, Princeton University, Princeton, New Jersey, USA; k Fogarty International Center, National Institutes of Health, Bethesda, Maryland, USA; l Department of Biosystems Science and Engineering, ETH Zurich, Zurich, Switzerland; m Centers for Disease Control and Prevention, Atlanta, Georgia, USA

## Abstract

Enterovirus A71 (EV-A71) is a major cause of hand, foot, and mouth disease (HFMD) and is particularly prevalent in parts of Southeast Asia, affecting thousands of children and infants each year. Revealing the evolutionary and epidemiological dynamics of EV-A71 through time and space is central to understanding its outbreak potential. We generated the full genome sequences of 200 EV-A71 strains sampled from various locations in Viet Nam between 2011 and 2013 and used these sequence data to determine the evolutionary history and phylodynamics of EV-A71 in Viet Nam, providing estimates of the effective reproduction number (R_e_) of the infection through time. In addition, we described the phylogeography of EV-A71 throughout Southeast Asia, documenting patterns of viral gene flow. Accordingly, our analysis reveals that a rapid genogroup switch from C4 to B5 likely took place during 2012 in Viet Nam. We show that the R_e_ of subgenogroup C4 decreased during the time frame of sampling, whereas that of B5 increased and remained >1 at the end of 2013, corresponding to a rise in B5 prevalence. Our study reveals that the subgenogroup B5 virus that emerged into Viet Nam is closely related to variants that were responsible for large epidemics in Malaysia and Taiwan and therefore extends our knowledge regarding its associated area of endemicity. Subgenogroup B5 evidently has the potential to cause more widespread outbreaks across Southeast Asia.

**IMPORTANCE** EV-A71 is one of many viruses that cause HFMD, a common syndrome that largely affects infants and children. HFMD usually causes only mild illness with no long-term consequences. Occasionally, however, severe infection may arise, especially in very young children, causing neurological complications and even death. EV-A71 is highly contagious and is associated with the most severe HFMD cases, with large and frequent epidemics of the virus recorded worldwide. Although major advances have been made in the development of a potential EV-A71 vaccine, there is no current prevention and little is known about the patterns and dynamics of EV-A71 spread. In this study, we utilize full-length genome sequence data obtained from HFMD patients in Viet Nam, a geographical region where the disease has been endemic since 2003, to characterize the phylodynamics of this important emerging virus.

## INTRODUCTION

Understanding the evolution and epidemiological dynamics of an infectious disease within and between countries where this disease is endemic is critical for predicting its emergence in new locations and to inform an effective public health response. Enterovirus A71 (EV-A71)-associated hand, foot and mouth disease (HFMD) is endemic in large parts of Southeast Asia, with a cyclical 2-to 3-year outbreak pattern (1–3). Notable outbreaks have occurred in 1998 in Taiwan (1,500,000 cases) (4), in 2008 in China (490,000 cases) (5), and in 2011 in Viet Nam (110,000 cases) (6). More recently, EV-A71 was detected in Cambodia, where it caused a major epidemic of severe HFMD with a 90% mortality rate (7). The increasing number of EV-A71 cases and the spread of the virus across Asia has raised major concerns about its pandemic potential (4, 7–10).

EV-A71 belongs to the species enterovirus A of the Enterovirus genus within the family Picornaviridae. Its genome comprises a single-stranded, positive-sense RNA molecule ∼7.5 kb in length. EV-A71 is thought to have diverged from coxsackievirus A16 around 1940 (11), and sequence analysis of the structural viral protein 1 (VP1) coding sequence indicates that it can be divided into three genogroups (A, B, and C). The latter two are further divided into several different subgenogroups, denoted B0 to B5 and C1 to C5.

EV-A71-related HFMD is most common in children and infants and is often associated with mild illness characterized by papulovesicular lesions on the hands, feet, orophyaryngeal mucosa, and buttocks (11). Occasionally, severe infections may arise, associated with neurological involvement, autonomic dysregulation, and often fatal cardiopulmonary complications. EV-A71 was first isolated from a patient with an infection of the central nervous system in California in 1969. Soon after, large outbreaks of EV-A71-associated encephalitis, with high mortality, were reported in Europe, North America, and Australia (12–16). However, it was not until 1973 that the first clear association between HFMD and EV-A71 was obtained during a large outbreak in Japan (17, 18). Outbreaks of EV-A71 have often been associated with switches between (sub)genogroups, occasionally accompanied by recombination events (8, 11, 19). For example, sequence data from Viet Nam obtained between 2005 and 2011 have shown that a subgenogroup switch from C5 to C4 occurred in 2011 (9, 20). Notably, this C4 subgenogroup has been associated with the ongoing epidemic of HFMD in China since 2008, as well as with the recent outbreak in Cambodia (7, 21).

Currently, there is no specific antiviral treatment available for EV-A71. Phase III trials of three different inactivated EV-A71 vaccines from China have recently been completed and have shown 95% protection rates against EV-A71-associated HFMD. Antibodies induced by natural infection with subgenogroup C4 or by vaccination with inactivated C4 induce broad cross-neutralizing activity for different EV-A71 subgenogroups, including genogroup B virus (22). Nevertheless, these vaccines are yet to be implemented, and it is unclear whether they also provide protection against the severe form of the disease (5, 23, 24) and to what extent they would shape the dynamics of (EV-A71-related) HFMD. In addition, enteroviruses commonly undergo extensive recombination. Such genetic exchanges can occur in, and even between, different serotypes, meaning that EV-A71 is highly variable both genetically and antigenically (25, 26).

Analysis of global VP1 sequences over a 4-decade period has shown that HFMD epidemics are likely to be driven in part by the migration of viruses between locations rather than continuous transmission within a locality (11). However, little is known about the patterns and dynamics of EV-A71 transmission at a local scale. We utilized full-length genome sequence data obtained from HFMD patients in Viet Nam to characterize viral phylodynamics. EV-A71-associated HFMD was first identified to be the source of an outbreak causing acute neurological disease in Viet Nam in 2003 (20) and still represents a major disease burden with an average of some 100,000 cases reported per year since 2011 (27). We report the population dynamics of EV-A71 within Viet Nam between 2011 and 2013, providing an estimate of temporal changes in the effective reproduction number (R_e_) of the infection through time as a more quantitative reflection of epidemiological dynamics. In addition, we examine the epidemiological dynamics of EV-A71 throughout Southeast Asia, where epidemics of EV-A71-associated HFMD have been established since 1973. Overall, these data provide a unique opportunity to investigate the phylodynamics of EV-A71 within a geographical region where it is endemic.

## MATERIALS AND METHODS

### Settings: studies, patients, and clinical samples.

The studies described here were conducted at three primary, secondary, and tertiary referral hospitals for infectious diseases, covering all provinces in Viet Nam. These included the Hospital for Tropical Diseases (HTD), Children's Hospital 1 (CH1), and Children's Hospital 2 (CH2). All of the hospitals are located in Ho Chi Minh City (HCMC) and serve a population of 42 million people. Patient enrollment was undertaken between August 2011 and December 2012 and between July 2013 and December 2013 as part of two different studies.

The first study was conducted at the Pediatric Intensive Care Unit (PICU) at HTD between August 2011 and December 2012, and at the Infectious Diseases Department and PICU at CH1 between September and December 2011. These departments only admitted HFMD patients with a clinical diagnosis of moderate to severe HFMD with neurological involvement. Swabs (including rectal/throat/vesicle swabs) were collected from anonymous HFMD children for routine diagnostics as part of standard patient management and placed in viral transport medium (28). After routine diagnostic analysis, any remaining swab material was stored at 4°C and transferred to the Oxford University Clinical Research Unit in HCMC, Viet Nam (OUCRU), where EV-A71 detection was performed using an in-house real-time reverse transcription-PCR targeting a VP1 fragment (9). Samples with a crossing point (Cp) value of ≤32 were then selected for whole-genome amplification and sequencing. Demographic and basic clinical information (including clinical grading on admission and at discharge) were also retrieved from the electronic database systems of the collaborating hospitals. In total, 250 samples (including 201 throat swabs, 47 rectal swabs, and 2 vesicle swabs) were included.

The second cohort was part of an ongoing observational study on HFMD patients of all severities conducted at the three hospitals described above. Patient enrollment began in July 2013 and is ongoing. This study reported the analysis of whole-genome sequencing efforts on 22 throat swabs collected at the Outpatient Clinic of CH2 and HTD, as well as the Infectious Diseases Department and PICU of CH2, between July and November 2013.

### Ethics statement.

The institutional review boards (IRBs) of HTD and CH1 in HCMC, Viet Nam, approved the whole-genome sequencing of residual swabs of anonymous HFMD patients. The observational study on HFMD was approved by the IRB of the collaborating hospitals and by the Oxford University Tropical Research Ethics Committee.

### RNA extraction, whole-genome amplification, and sequencing.

Whole-genome amplification and sequencing of the viral genomes were done either at the J. Craig Venter Institute (Rockville, MD) for the first cohort or at OUCRU for the second cohort.

### First study.

A total of 200 μl of viral clinical material was combined with 600 μl of Zymo research viral RNA buffer containing 0.1% 2-mercaptoethonol. Viral RNA was extracted using a ZR-96 viral RNA kit (Zymo Research Corp., Irvine, CA). Full-length cDNA was generated using the SuperScript III First-Strand Synthesis SuperMix (Thermo Fisher Scientific, Waltham, MA) and oligo(dT) (45°C for 15 min, 50°C for 50 min, and 55°C for 15 min). Due to the complex structure of the EV71 internal ribosome site, two DNA fragments (short and long) were PCR amplified for each virus. A short fragment (∼640 bp) was generated with a Q5 Hot Start high-fidelity DNA polymerase reaction system (New England BioLabs, Ipswich, MA) and the primers EV71-1F (TTAAAACAGCCTGTGGGTT) and EV71-641R (GGCCAATCCAATAGCTATATG) at concentrations of 10 μM each. Next, 3 μl of cDNA was added to 7 μl of Q5 reaction mixture and subjected to the following thermal cycling program: 98°C for 3 min (98°C for 20 s, 62°C for 30 s, and 72°C for 1 min + 5 s/cycle) for 40 cycles, followed by 72°C for 10 min and then a 4°C hold. A long fragment (∼7 kb) was generated using a Phusion high-fidelity PCR kit (New England BioLabs, Ipswich, MA) and the primers EV71-473F (AATGCGGCTAATCCYAACT) and EV71-7405R (GCTATTCTGGTTATAACAAATTTACC) at concentrations of 10 μM each. Then, 4 μl of cDNA was added to a 21-μl reaction mixture, which was then subjected to the following thermal cycler program: 98°C for 3 min (98°C for 30 s, 62°C for 30 s, and 72°C for 6 min + 5 s/cycle) for 35 cycles, followed by 72°C for 10 min and then a 4°C hold. The short amplicons were detected and quantitated on a QIAxcel capillary electrophoresis platform, while the long amplicons were detected via agarose gel electrophoresis and quantitated using Sybr green and Gen5 software.

For each virus sample with two amplicons, 15 ng of the short amplicon and 150 ng of the long amplicon were pooled. Illumina libraries were prepared from the pooled amplicons using the Nextera DNA sample preparation kit (Illumina, Inc., San Diego, CA) with half reaction volumes. Briefly, 25 ng of pooled DNA amplicons were tagmented at 55°C for 5 min. Tagmented DNA was cleaned with a ZR-96 DNA Clean & Concentrator kit (Zymo Research Corp., Irvine, CA) and eluted in 25 μl of resuspension buffer. Illumina sequencing adapters and barcodes were added to tagmented DNA via PCR amplification: 20 μl of tagmented DNA was combined with 7.5 μl of Nextera PCR master mix, 2.5 μl of Nextera PCR Primer Cocktail, and 2.5 μl of each index primer (Integrated DNA Technologies, Coralville, IA) for a total volume of 35 μl per reaction mixture. Thermal cycling was performed with 5 cycles of PCR according to the Nextera DNA sample preparation kit protocol (3 min at 72°C, denaturation for 10 s at 98°C, annealing for 30 s at 63°C, and extension for 3 min at 72°C) to create a dual indexed library for each sample. After PCR amplification, 10 μl of each library was pooled into a 1.5-ml tube, and the pool was cleaned twice with Ampure XP reagent (Beckman Coulter, Inc., Brea, CA) to remove all leftover primers and small DNA fragments. The first cleaning used a 1.2× volume Ampure reagent, while the second cleaning used a 0.6× volume Ampure reagent. The two pools were sequenced on an Illumina MiSeq v2 instrument with 300-bp paired-end reads.

The sequence reads from the Illumina data were sorted by barcode, trimmed, and *de novo* assembled using CLC Bio's (Qiagen, Hilden, Germany) the clc_novo_assemble program, and the resulting contigs were searched against custom full-length EV-A71 nucleotide databases to find the closest reference sequence for each virus. All sequence reads were then mapped to the selected reference EV-A71 virus using CLC Bio's clc_ref_assemble_long program.

### Second study.

Total nucleic acid was isolated from 140 μl of clinical material using the QIAamp viral RNA kit (Qiagen), recovered in 50 μl of elution buffer (provided with the kit), and was immediately stored at −80°C for the subsequent whole-genome amplification step. Whole-genome amplification and sequencing was undertaken as previously described using in-house designed PCR primers and the Miseq system (Illumina), respectively (29). The reads obtained were processed to remove PCR primers using CLC Genomics Workbench (Qiagen). Sequence assembly was performed using the Genieous 7.1.3 software package utilizing a reference-based mapping tool (i.e., the consensus sequence was obtained by mapping individual reads of each sample to a reference sequence).

### Recombination analysis.

Because enteroviruses are known for their ability to undergo extensive recombination (30), we determined the frequency and occurrence of this process in the 200 whole genomes sequenced in this study by using the genetic algorithm recombination detection (GARD) method available at the Datamonkey webserver (31). This analysis used the Hasegawa-Kishino-Yano (HKY) model of nucleotide substitution and default parameters in all other cases.

### Phylogeny and phylogeography of EV-A71.

The phylogenetic relationships of EV-A71 within Southeast Asia were estimated from a total of 1,176 complete VP1 sequences (891 nucleotides [nt]), including the 200 sequences from Viet Nam obtained in the present study. The data set also comprised 976 randomly selected sequences downloaded from GenBank, sampled between 1997 and 2013. Sequences were aligned in Geneious (v7.1.3) using the multiple-sequence alignment tool, MAFFT. Phylogenetic inference utilized the maximum-likelihood (ML) method available in RAxML (v7.2.8), applying the general time reversible (GTR) nucleotide substitution model with a gamma (Γ) distribution of among-site rate variation. Support for individual nodes was assessed using a bootstrap procedure with 100 replicates.

This EV-A71 data set was also used to investigate the phylogeographic history of EV-A71 in Southeast Asia. To this end, the data were grouped into 10 discrete country (or autonomous regions) locations within Southeast Asia (China, Hong Kong, Japan, South Korea, Malaysia, Philippines, Singapore, Taiwan, Thailand, and Viet Nam). For this analysis we used a time-aware coalescent Bayesian skyride model available in BEAST (32) (v1.8), which utilizes Gaussian Markov random field smoothing prior to estimate the changes in relative genetic diversity over time (33). The HKY model of nucleotide substitution was used (determined by JModelTest [version 2.1.4] to be the best-fit model), along with an uncorrelated relaxed molecular clock and a uniform distributed clock rate prior of 0.004. Using the Bayesian Markov Chain Monte Carlo framework, 100 million steps were run, sampling every 10,000 and removing 10% as burn-in. Convergence was assessed using Tracer (v1.4) (34), and effective sample size (ESS) values above 200 were accepted. A maximum clade credibility (MCC) tree was summarized with TreeAnnotator (available in the BEAST package) and visualized in Figtree (v1.4).

To determine whether there was more clustering of EV-A71 by place of sampling than expected by chance alone, we utilized two phylogeny-trait association tests: the association index (AI) and parsimony score (PS), both of which are available in the BaTS package (35). The maximum-clade statistic was also used to determine which individual locations showed the strongest spatial clustering. Phylogenetic uncertainty in the data was incorporated through the use of the posterior distribution of trees (determined from the BEAST analysis described above) with 1,000 random permutations of sampling locations undertaken to create a null distribution for each statistic.

We also investigated phylogeography at a local level within Viet Nam. Of the 200 whole-genome samples successfully sequenced, 190 included the place (province) in which the patient was registered (and we assume this was where the disease was contracted). We analyzed these samples from 18 discrete provinces in Viet Nam, using the phylogeny-trait association tests described above (using both whole genomes and VP1). Unfortunately, the relatively small sample size, together with the collection methods of the different cohorts, prevented us from exploiting clinical information associated with the disease in our analyses.

### Phylodynamics.

**FIG 1:**
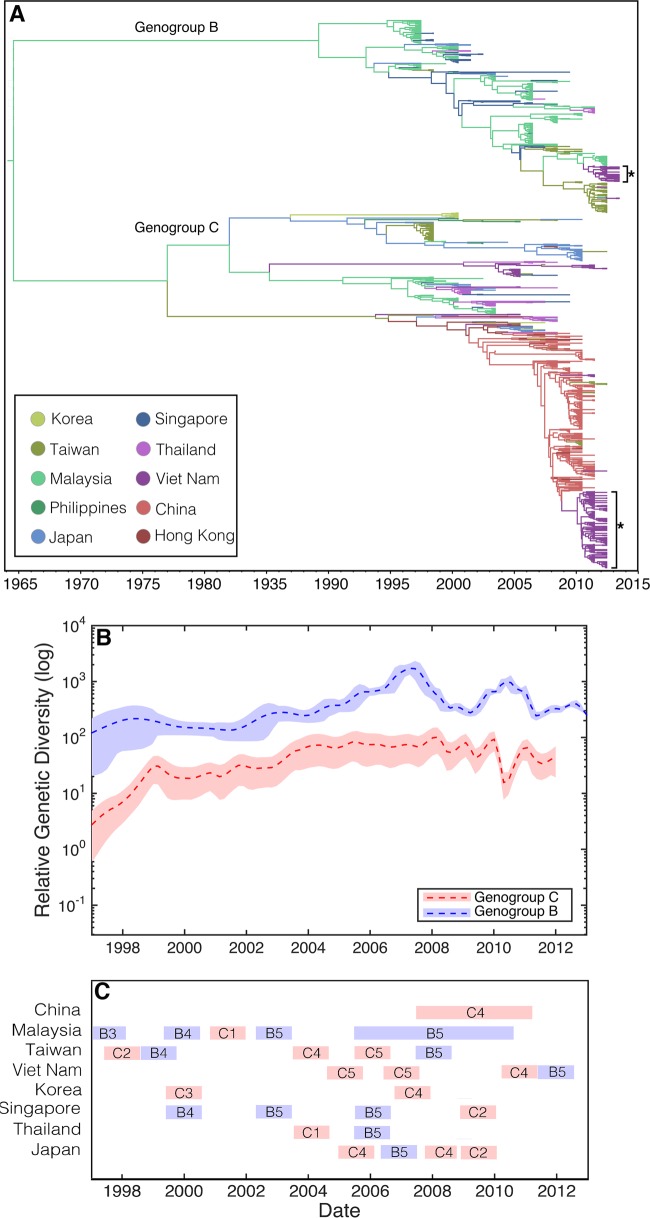
(A) MCC tree of 1,176 complete VP1 sequences. The phylogeny includes 200 sequences from Viet Nam, as well as 976 randomly selected sequences from GenBank, sampled from 10 discrete locations in Southeast Asia (China, Hong Kong, Japan, South Korea, Malaysia, Philippines, Singapore, Taiwan, Thailand, and Viet Nam) between 1997 and 2013. Branches are color-coded according to the location of sampling. Asterisks indicate the two major subgenogroups from Viet Nam sampled in the present study. (B) Bayesian skyride plots, illustrating the relative genetic diversity of genogroups B (blue) and C (red) through time. A dashed line indicates the mean while shaded areas show the upper and lower 95% HPD values. (C) The temporal distribution of the different subgenogroups circulating in the region (partially adapted from Fig. 2.2 in reference 54). The data are from countries with enhanced surveillance since 1997.

To determine the population dynamics of EV-A71 in Viet Nam, we analyzed whole-genome (7,360 nt) sequences, together with their exact date (day) of sampling, for the two main viral lineages circulating in Viet Nam, representing subgenogroup B5 (*n* = 38) and subgenogroup C4 (*n* = 154) (these sequences are marked with an asterisk in Fig. 1A). Lineages were compared using the same Bayesian skyride method described above (using both whole-genome sequences and VP1), which enables us to reveal changes in relative genetic diversity over time.

In addition, we estimated temporal changes in R_e_ for subgenogroups C4 and B5 in Viet Nam using the birth-death skyline model (BDSKY) (36) available in BEASTii (v2.1.3). For each analysis, we utilized the same methods as described above: the HKY model of nucleotide substitution, an uncorrelated relaxed molecular clock, and a uniform distributed clock rate prior of 0.004. We also assumed that the proportion of individuals being sampled was <5% of the total infected population. This assumption was based on the assessment that <5% of the total number of symptomatic cases were subjected to virological diagnosis and that the vast majority of EV-A71 infections are asymptomatic (37). We allowed the sampling rate to change over time, reflecting the heterogeneity of collections in the study, while assuming that the rate at which patients recover remained constant. For the rate at which individuals recover, which we assume to coincide with becoming noninfectious, we used a lognormal distribution prior [logN(3.415,0,2)], which translates to the sum of the incubation (∼5 days) and infectious period (∼7 days) (38) to center around 12 days.

### GenBank accession numbers.

The genome sequences of EV-A71 obtained in the present study have been deposited on GenBank under accession numbers KJ686127 to KJ686308 and KP691643 to KP691664.

## RESULTS

### EV-A71 genome sequences.

In total, 200 full (consensus) genome sequences of EV-A71 were successfully generated as part of this project: 178 by sequencing efforts undertaken at the J. Craig Venter Institute funded by a U.S. National Institutes of Health/National Institute of Allergy and Infectious Disease Genome Sequencing Center contract and an additional 22 complete genome sequences through an in-house sequencing pipeline at OUCRU.

### Recombination in Vietnamese EV71.

Our recombination analysis (GARD) detected a single breakpoint (*P* < 0.01) in the 2C gene sequence (nucleotide position 4554). Accordingly, ML phylogenetic trees were then inferred on either side of this breakpoint, and these revealed that sequence ID:0_023 (collected from Binh Duong province on 21 August 2013) had experienced a minor change of topological position within the subgenogroup B5 clade. Because of the minimal phylogenetic movement, this sequence was retained in the data set. No other recombination events were detected, which is also supported by the very tight clustering within each of the C4, C5 and B5 subgenogroups.

### Phylogeny and phylogeography.

Phylogenetic analysis of 1,176 complete VP1 gene sequences sampled between 1997 and 2013 revealed the existence of two major genogroups, B and C (see Fig. S1 in the supplemental material). The majority of samples obtained from Viet Nam in this study fell into two lineages: 154 in subgenogroup C4 and 38 in subgenogroup B5, while the remaining 8 sequences clustered in subgenogroup C5.

Phylogeographic analysis revealed significant spatial clustering by sampling location (i.e., country or autonomous region): AI (*P* = 0) and PS (*P* = 0). These results were summarized using an MCC tree in which branches were colored according to sampling location (Fig. 1A). Notably, both genogroups exhibited a varied, multicountry distribution. Subgenogroup C4 viruses in Viet Nam were closely related to those from China and Hong Kong and, given these data, likely originated from there. In contrast, genogroup B predominantly contained samples from Malaysia and Taiwan, to which the small subgenogroup B5 samples from Viet Nam were closely related. Several viruses from Viet Nam sampled first in 2005 (20) and then in 2011 and 2012 (the present study) were found to be members of subgenogroup C5.

**FIG 2:**
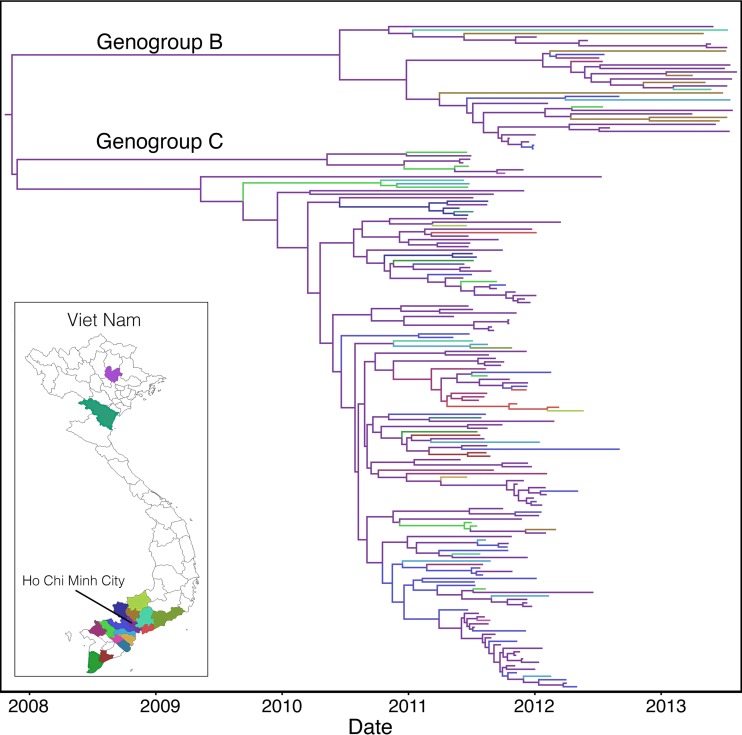
MCC tree of 190 whole-genome EV-A71 sequences sampled from 18 provinces in Viet Nam between 2011 and 2013. Branches are color-coded according to location of sampling.

The phylogeography of EV-A71 within Viet Nam was similarly examined by considering the 18 discrete provinces from which the samples originated. Although we found more spatial clustering by sampling location than expected by chance (AI, *P* = 0; PS, *P* < 0.01), the relatively small sample size in certain provinces, as well the intrinsic biases in sampling protocols, prohibited further spatial analysis (Fig. 2). Nevertheless, this analysis showed some fluidity within Viet Nam, as is evident from the phylogenetic tree, compared to relatively infrequent movement of viruses at the international level. This may in part be due to the small geographic region in southern Viet Nam from which the majority of samples were taken (only two sequences originated from northern provinces, Thai Nguyen and Thanh Hoa, with the remainder sampled from southern Viet Nam). To account for the possible impact of recombination on our phylogeographic inferences, we repeated this analysis on the VP1 gene alone, which resulted in very similar patterns to observed with the complete genome data (see Fig. S2A in the supplemental material).

### EV-A71 phylodynamics.

Bayesian skyride plots of genogroups B and C depict changes in relative genetic diversity over time for EV-A71 across Southeast Asia as a whole (Fig. 1B). Although these results need to be treated with caution because of the occurrence of population subdivision by country, it is clear from this analysis that irregular cycles of relative genetic diversity were observed, often sharply increasing then decreasing. Some of these irregular cycles coincided with large EV-A71 epidemics, most notably the 2006 B5 outbreak that affected Malaysia, Thailand, and Singapore (39) (Fig. 1C).

**FIG 3:**
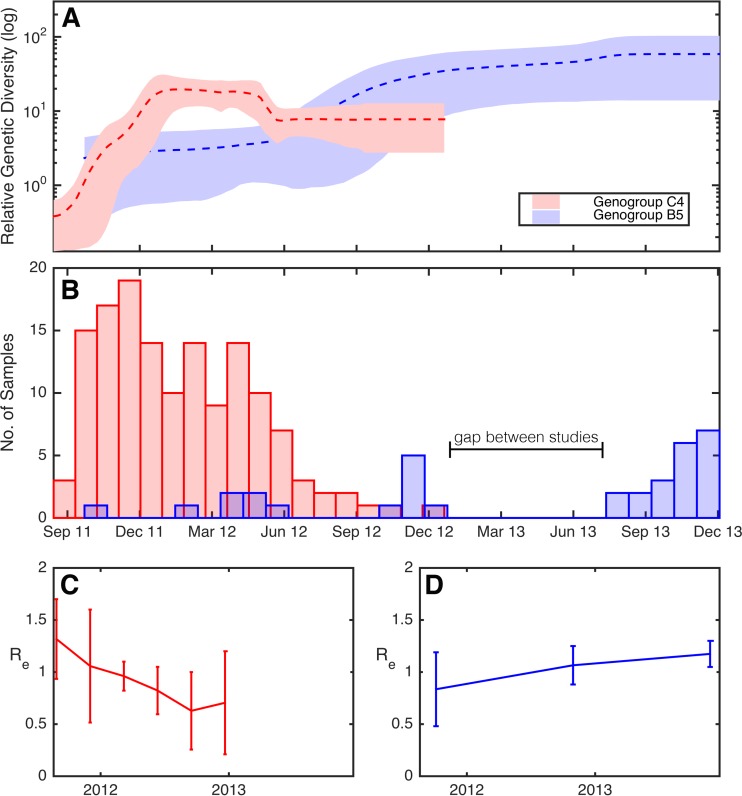
(A) Bayesian skyride plots showing changing levels of relative genetic diversity over time of the two Viet Nam lineages indicated by asterisks in Fig. 1A. Two skyride plots are shown: one from subgenogroup B5 (blue), and the other from subgenogroup C4 (red). A dashed line indicates the mean while the shaded area shows the upper and lower 95% HPD values. (B) Sampling prevalence of subgenogroup C4 (red) and B5 (blue) over the study period. Changing values of R_e_ over time for EV-A71 subgenogroups C4 (C) and B5 (D), estimated using a serial-sampled birth-death model. The line plot shows the median estimate of R_e_, while 95% HPD values are given by error bars.

Two distinct lineages from Viet Nam—subgenogroups B5 and C4—were analyzed in more detail (sequences indicated on Fig. 1A with asterisks). In particular, we again used the Bayesian skyride analysis to reveal temporal changes in genetic diversity (Fig. 3A). Notably, the skyride plot captures the rapid increase in genetic diversity of subgenogroup C4 at the end of 2011 before peaking at the beginning of 2012, followed by a brief decline from May to June and eventually a plateau. In contrast, the relative genetic diversity of subgenogroup B5 increased markedly over the same time period, seemingly replacing subgenogroup C4 at the end of 2012, and continued to increase during 2013. This observation is consistent with a subgenogroup switch from C4 to B5 during 2012. Indeed, all samples collected during 2013 in Viet Nam were exclusively subgenogroup B5 (Fig. 3B). This analysis was also undertaken using the VP1 gene sequences and captured a very similar pattern, indicating that it has not been adversely affected by recombination (see Fig. S2B in the supplemental material).

Finally, we obtained R_e_ estimates for subgenogroup C4 in Viet Nam using a phylogenetic (birth-death) approach. Notably, the BDSKY analysis provided clear evidence for temporal changes of R_e_ during the 2011-2013 outbreak. Specifically, there was a decrease in the R_e_ of subgenogroup C4 in Viet Nam in 2012 (Fig. 3C). Concomitantly, we observed a slight increase in R_e_ of subgenogroup B5 during 2012 and, importantly, R_e_ remained >1 at the end of the study period (Fig. 3D). The total duration of incubation and infectiousness estimated under this approach was 17.9 days (95% HPD, 11.7 to 25.9) for C4 and 19.8 days (95% HPD, 13.3 to 28.0) for B5. Again, we repeated this analysis using the VP1 gene sequences, which resulted in a similar trend (see Fig. S2C and D in the supplemental material).

## DISCUSSION

This study documented the evolutionary history of the main genogroups of EV-A71 circulating in Viet Nam and throughout Southeast Asia. Our analysis of the overall population genetic history of EV-A71 revealed major fluctuations in genetic diversity through time, coinciding with large and varied epidemics, thereby supporting previous observations (11). Notably, EV-A71 genetic diversity remained relatively high even between large outbreaks, suggesting that the virus was likely sustained in the local population.

Our phylogenetic analysis revealed the presence of two main genogroups, B and C, in the Southeast Asian region. While both genogroups exhibited a multicountry distribution, there was also strong clustering by sampling location. More refined analysis revealed that genogroups C4 and B5 were the two major viruses present in Viet Nam during the course of this study. Our phylogeographic analysis revealed that subgenogroup C4 likely originated from the large and ongoing outbreak in China, while B5 is closely related to sequences sampled from Malaysia and Taiwan, and likely entered Viet Nam circa 2010 (2 to 3 years after that of subgenogroup C4). Indeed, genogroup B5 was the cause of the major Malaysian (Sarawak) outbreak in 2006 (40), and its emergence in Taiwan in 2008 resulted in the region's largest outbreak of HFMD in 11 years (41). We also found a small lineage of subgenogroup C5 viruses in Viet Nam that were closely related to a previous small outbreak in 2005 (also in Viet Nam) (20), suggesting that C5 virus may have been in continual circulation at low prevalence over this period.

During 2011-2012, a large outbreak of EV-A71 associated HFMD occurred in Viet Nam followed by ongoing circulation, but with fewer (severe) cases, in 2013-2014 (42). By examining population dynamics at a local level within Viet Nam, our data suggested that a rapid genogroup switch from C4 to B5 likely took place at the end of 2012. Genogroup switching has been seen elsewhere during previous outbreaks of EV-A71-associated HFMD (8, 11, 19, 43, 44), including in Malaysia, Japan, Viet Nam, and Taiwan (8, 20). For example, between 2009-2012, the dominant genogroup in Taiwan appeared to have changed twice: from B to C and then later from C to B (41).

EV-A71 exists as a single serotype, as measured by hyperimmune animal antiserum, yet results from human studies on antigenic variations among subgenogroups have been complex and inconsistent. Antigenic cartography has revealed clear antigenic differences among (sub)genotypes, with B5 isolates from the 2008 outbreak in Taiwan comprising a distinct group on the antigenic map and possessing an Asp164 amino acid residue in VP1 that distinguished it from other genogroup B viruses (41, 45, 46). In contrast, antibodies from C4 vaccines, as well as from children naturally infected with a variety of EV-A71 subgenogroups, cross-neutralized a broad range of genogroup B and C viruses, including B5 (22, 47). These sera were all taken just weeks after vaccination or infection and it is possible that the initial wide spectrum of the cross-neutralizing immune response narrows over time to become more focused on the vaccine/infecting strain, as previously described in dengue and influenza virus infections (48, 49). Interestingly, however, sera from healthy individuals in Japan, where EV-A71 is endemic and caused outbreaks every 3 years, also showed a broad spectrum of neutralizing activity (50), implying that cross-neutralizing antibodies are long lasting but may also be caused by (multiple) infections with different closely related enterovirus serotypes and/or EV-A71 subgenogroups.

The genogroup switch from subgenogroup C4 to B5 in Viet Nam may imply that the circulation of C4 did not provide lasting cross-protection to B5 infection and tentatively suggests that host immunity may drive genogroup switching. However, this idea clearly needs to be tested experimentally, and it was notable that the switch to B5 was not associated with another large outbreak as in 2011 and 2012. This may be explained by partial residual herd immunity against B5 after C4 circulation, by depletion of the susceptible population of children under five after 2 years of hyperendemicity of C4, or by other phenotypic changes in the virus that have accumulated as a consequence of its ongoing genetic evolution. Alternatively, it is possible that the C4 and B5 subgenogroups are in fact cross-reactive but were infecting different population groups within Viet Nam. Further research is evidently needed to study the spectrum and duration of cross-neutralizing activity of EV-A71 antibodies among vaccines and recovered patients, and to determine to what extent the implementation of a single subgenogroup vaccine would shape the dynamics of HFMD.

Our estimate of R_e_ through time showed a decrease in the transmission of subgenogroup C4 in Viet Nam between 2011 and 2012. This pattern is consistent with the reduction in C4 cases, as well as with the decrease in genetic diversity observed during the latter half of this study. In contrast, we observed a rise in the reproductive number of the B5 subgenogroup, which remained >1 at the end of 2013. Previously, R_e_ for EV-A71 has been determined using incidence data obtained from laboratory-confirmed HFMD outbreaks, with median estimates ranging from 1.4 (51) to 5.5 (38) and sometimes higher (up to 10 to 15 [52]). Our temporal and gene sequence-based estimates are at the lower end of this range, which may be due to the fact that our analyses are focused on separate subgenogroups, instead of the overall EV-A71 virus population as the case with incidence-based measures. The relatively short sampling time frame used here may also have produced an incomplete picture. For instance, we have apparently captured data concerning only the decline phase in C4 transmission, as well as data concerning only the initial phase of the B5 introduction into Viet Nam. Of note, it has been hypothesized that emerging subgenogroups may cryptically circulate in the community at low prevalence for years and cause mild infection prior to emergence (11). However, it is surprising that our estimates of R_e_ are low (albeit with the range previously reported for EV-A71), given the seemingly rapid and large epidemic experienced in Viet Nam, although they are robust to a variety of prior distributions for the input parameters. Nonetheless, our observational data on patients enrolled into the ongoing cohort showed that the overall prevalence of EV-A71 is currently low (data not shown). Although a major advantage of these phylodynamic methods is that they allow population dynamic estimations from sequences when other data are lacking, further research, such as subgenogroup-specific prevalence data, will be valuable in confirming these results.

It is worth noting that sampling time frame, dissimilarities in recruitment protocols, and the brief hiatus between studies may have had some affect on the results presented here. In particular, our observation of subgenogroup switching was concomitant with major changes in study design. Despite this short-coming, Bayesian inference of past evolutionary events using genome sequence data is capable of accounting for such discrepancies in sampling frequency. In addition, this study utilizes data from an ongoing recruitment effort and thus paves the way for further, more in-depth analysis on the phylodynamics of EV-A71 in Viet Nam and throughout Southeast Asia.

Finally, our estimates of the infectious period were slightly higher than previously estimated. It is generally assumed that infectiousness lasts for approximately 7 days (38). However, several studies suggest a much longer period, lasting several weeks to months (53), albeit without clear evidence of virus transmission. Accurately predicting the number of contacts and the probability of transmission is often difficult to establish from case data alone, in which case our estimates of a longer infectious period may be plausible.

## Supplementary Material

Supplemental material
