# Supplementary material for: Phylodynamics of Enterovirus A71-Associated Hand, Foot, and Mouth Disease in Viet Nam
Source: J Virol. 2015 Jun 17;89(17):8871–9. doi: 10.1128/JVI.00706-15 (PMC4524079; doi:10.1128/JVI.00706-15)
Supplement: Supplemental material [file supp_89_17_8871__index.html]

Phylodynamics of Enterovirus A71-Associated Hand, Foot, and Mouth Disease in Viet Nam — Supplemental material 

# Phylodynamics of Enterovirus A71-Associated Hand, Foot, and Mouth Disease in Viet Nam

## Supplemental material

- Supplemental file 1 -

  Fig. S1 (ML phylogeny of the complete VP1 gene, including sequences sampled within Viet Nam obtained from this study and those from Southeast Asia downloaded from GenBank.)

  Fig. S2 ((A) MCC tree of 190 VP1 gene EV-A71 sequences sampled from 18 provinces in Viet Nam between 2011 and 2013. (B) Bayesian skyride plots utilizing the VP1 gene, showing changing levels of relative genetic diversity over time of two Viet Nam lineages. (C and D) Changing values of Re over time using the VP1 gene of EV-A71 subgenogroup C4 and subgenogroup B5, estimated using a serially sampled birth-death model.)

  PDF, 2.5M
