# Supplementary material for: Phylodynamics of Enterovirus A71-Associated Hand, Foot, and Mouth Disease in Viet Nam
Source: J Virol. 2015 Jun 17;89(17):8871–9. doi: 10.1128/JVI.00706-15 (PMC4524079; doi:10.1128/JVI.00706-15)
Supplement: Supplemental material [file JVI.00706-15_zjv999090699so1.pdf]

Supplementary Information

Supplementary Figure 1. ML phylogeny of the complete VP1 gene, including sequences sampled within Viet Nam obtained from this study (n=200; shown in red), as well as those from Southeast Asia downloaded from GenBank (n=976). The different genogroups and subgenogroups are marked accordingly. All horizontal branch lengths are drawn to a scale of nucleotide substitutions per site, and the tree is rooted between the B and C genogroups. Bootstrap analysis was performed using 100 replicates (only those >70% are also shown).

Supplementary Figure 2. **(A)** MCC tree of 190 VP1 gene EV-A71 sequences sampled from 18 provinces in Viet Nam between 2011-2013. Branches are color-coded according to location of sampling. **(B)** Bayesian skyride plots utilizing the VP1 gene, showing changing levels of relative genetic diversity over time of the two Viet Nam lineages indicated by asterisks in Figure 1A. Two skyride plots are shown: one from subgenogroup B5 (blue), and the other from subgenogroup C4 (red). A dashed line indicates the mean while the shaded area shows the upper and lower 95% HPD values. **(C)** Changing values of R_e over time using the VP1 gene of EV-A71 subgenogroup C4 and subgenogroup B5 **(D)**, estimated using a serial-sampled birth-death model. The line plot shows the median estimate of R_e, while 95% HPD values

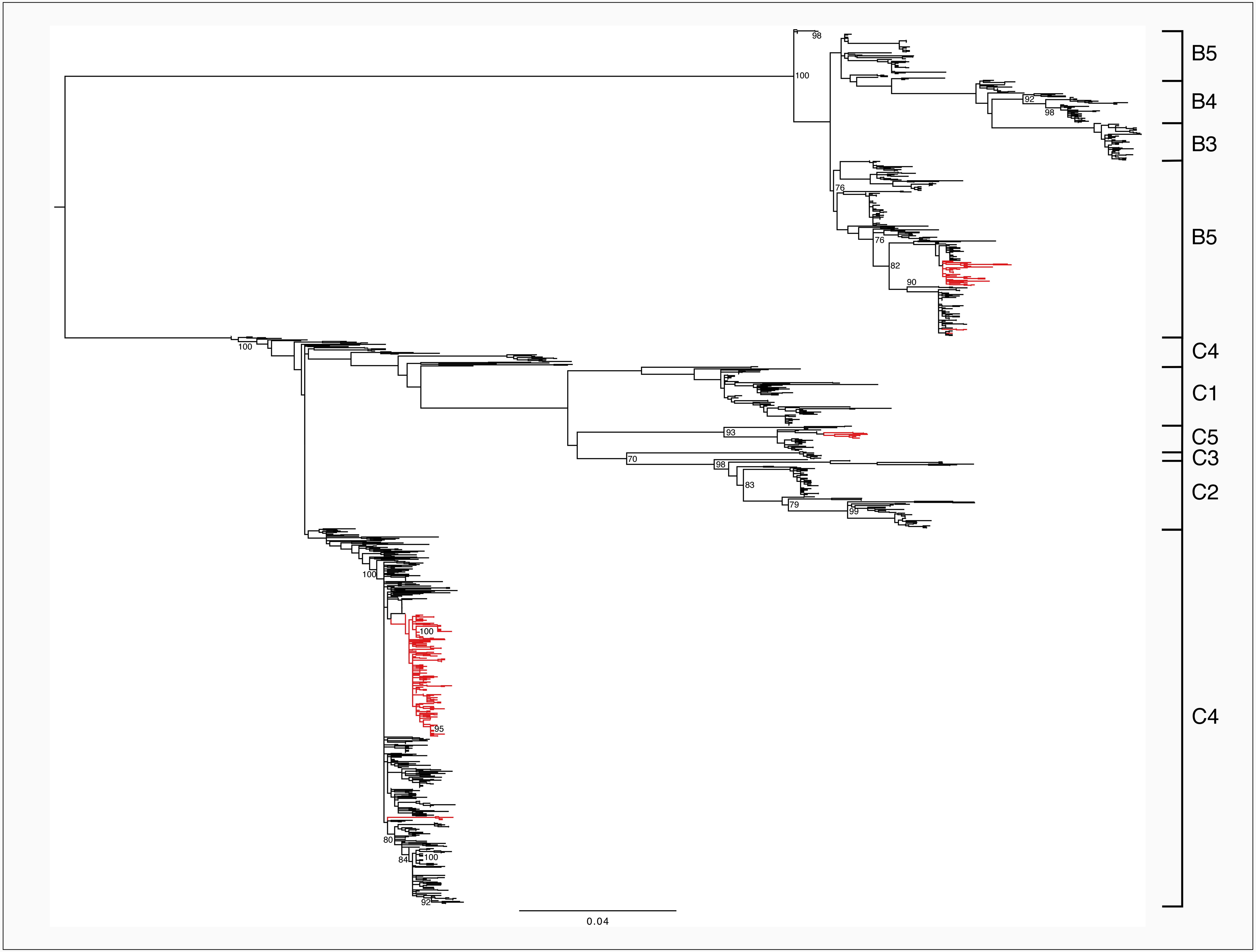

B5
B4
B3
B5
C4
C1
C5
C3
C2
C4
0.04

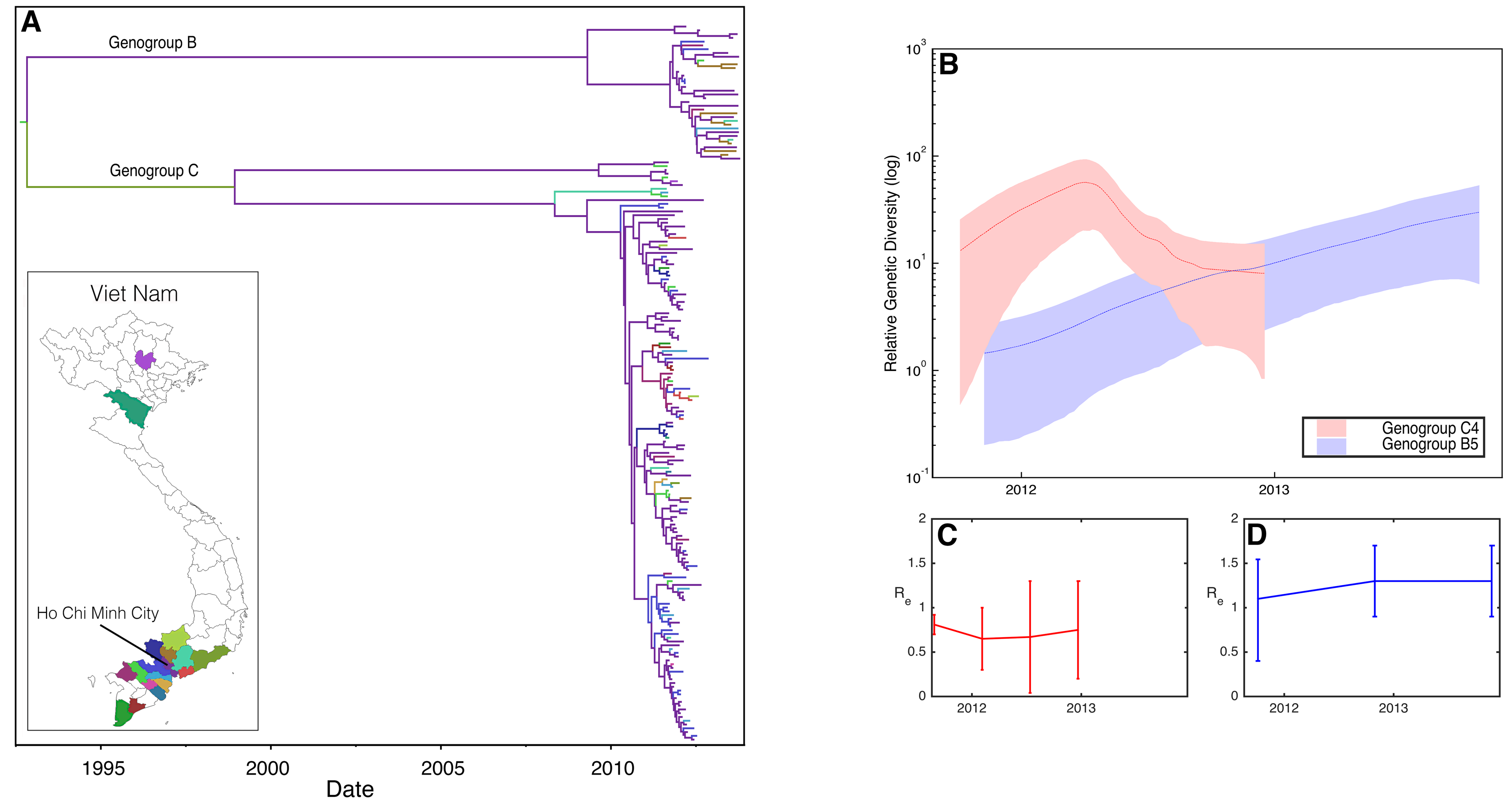
A
Genogroup B
Genogroup C
Viet Nam
Ho Chi Minh City
1995
2000
2005
2010
Date
B
Relative Genetic Diversity (log)
10^3
10^2
10^1
10^0
10^{-1}
2012
2013
Genogroup C4
Genogroup B5
C
R_e
2
1.5
1
0.5
0
2012
2013
D
R_e
2
1.5
1
0.5
0
2012
2013
